# The diagnostic accuracy of lung ultrasound to determine PiCCO-derived extravascular lung water in invasively ventilated patients with COVID-19 ARDS

**DOI:** 10.1186/s13089-023-00340-7

**Published:** 2023-10-02

**Authors:** Leila N. Atmowihardjo, Job R. Schippers, Mark E. Haaksma, Marry R. Smit, Harm J. Bogaard, Leo Heunks, Nicole P. Juffermans, Marcus J. Schultz, Henrik Endeman, Patricia van Velzen, Pieter R. Tuinman, Jurjan Aman, Lieuwe D. J. Bos

**Affiliations:** 1 Intensive Care, Amsterdam UMC Location University of Amsterdam, Meibergdreef 9, Amsterdam, The Netherlands; 2 Department of Pulmonology, Amsterdam UMC Location Vrije Universiteit Amsterdam, Boelelaan 1117, Amsterdam, The Netherlands; 3 Intensive Care, Amsterdam UMC Location Vrije Universiteit Amsterdam, Boelelaan 1117, Amsterdam, The Netherlands; 4 Intensive Care, Erasmus University Medical Center, Doctor Molewaterplein 40, Rotterdam, The Netherlands; 5 Mahidol Oxford Tropical Medicine Research Unit (MORU), Mahidol University, Bangkok, Thailand; 6 Nuffield Department of Medicine, University of Oxford, Oxford, UK; 7 Dijklander Hospital Location Purmerend, Intensive Care, Waterlandlaan 250, Purmerend, The Netherlands; 8 Amsterdam Leiden IC Focused Echography, Amsterdam, The Netherlands; 9 Laboratory of Translational Intensive Care, Erasmus University, Rotterdam, the Netherlands; 10 Department of Intensive Care Medicine, Amsterdam University Medical Center, Location AMC, Meibergdreef 9, Room G3-228, 1105 AZ Amsterdam, The Netherlands; 11 Department of Intensive Care, Radboud University Medical Center, Nijmegen, The Netherlands

**Keywords:** Ultrasonography, Thermodilution, Extravascular lung water, Pulmonary edema, COVID-19, Respiratory distress syndrome, Intensive care units

## Abstract

**Background:**

Lung ultrasound (LUS) can detect pulmonary edema and it is under consideration to be added to updated acute respiratory distress syndrome (ARDS) criteria. However, it remains uncertain whether different LUS scores can be used to quantify pulmonary edema in patient with ARDS.

**Objectives:**

This study examined the diagnostic accuracy of four LUS scores with the extravascular lung water index (EVLWi) assessed by transpulmonary thermodilution in patients with moderate-to-severe COVID-19 ARDS.

**Methods:**

In this predefined secondary analysis of a multicenter randomized-controlled trial (InventCOVID), patients were enrolled within 48 hours after intubation and underwent LUS and EVLWi measurement on the first and fourth day after enrolment. EVLWi and ∆EVLWi were used as reference standards. Two 12-region scores (global LUS and LUS–ARDS), an 8-region anterior–lateral score and a 4-region B-line score were used as index tests. Pearson correlation was performed and the area under the receiver operating characteristics curve (AUROCC) for severe pulmonary edema (EVLWi > 15 mL/kg) was calculated.

**Results:**

26 out of 30 patients (87%) had complete LUS and EVLWi measurements at time point 1 and 24 out of 29 patients (83%) at time point 2. The global LUS (*r* = 0.54), LUS–ARDS (*r* = 0.58) and anterior–lateral score (*r* = 0.54) correlated significantly with EVLWi, while the B-line score did not (*r* = 0.32). ∆global LUS (*r* = 0.49) and ∆anterior–lateral LUS (*r* = 0.52) correlated significantly with ∆EVLWi. AUROCC for EVLWi > 15 ml/kg was 0.73 for the global LUS, 0.79 for the anterior–lateral and 0.85 for the LUS–ARDS score.

**Conclusions:**

Overall, LUS demonstrated an acceptable diagnostic accuracy for detection of pulmonary edema in moderate–to–severe COVID-19 ARDS when compared with PICCO. For identifying patients at risk of severe pulmonary edema, an extended score considering pleural morphology may be of added value.

*Trial registration*: ClinicalTrials.gov identifier NCT04794088, registered on 11 March 2021. European Clinical Trials Database number 2020–005447-23.

**Supplementary Information:**

The online version contains supplementary material available at 10.1186/s13089-023-00340-7.

## Background

The accumulation of protein-rich fluid in the interstitial and alveolar space is a central hallmark of Acute Respiratory Distress Syndrome (ARDS) [1]. The extent of pulmonary edema influences the course and severity of respiratory insufficiency [2] and outcomes of patients with ARDS [3, 4]. Quantification of pulmonary edema aids in monitoring disease course and guides clinical decision-making [5–8], for instance regarding fluid management and the initiation of invasive ventilation. However, accurate quantification is not a simple task. Pulmonary edema can be assessed by several methods, including computed tomography (CT) [9, 10], chest X-ray [3] and pulse contour cardiac output (PiCCO) transpulmonary thermodilution. The former two techniques use ionizing radiation and CT requires patient transport. Extravascular lung water index (EVLWi) measurement by PiCCO requires arterial and central venous cannulation [11, 12]. Lung ultrasound (LUS) is a non-invasive imaging method that can be used to assess edema [7, 13, 14], as well as pleural effusions, consolidations, pneumothorax and pleural abnormalities [15, 16].

PiCCO-derived EVLWi is a validated, quantitative measure of pulmonary edema in ARDS [11, 17–19]. Recent years have seen an effort to quantify pulmonary edema using LUS [7, 14, 15] using B-lines, ultrasonographic artifacts thought to arise from the change in acoustic impedance between aerated and non-aerated tissue [20]. Evidence of the correlation of LUS with EVLWi on the Intensive Care Unit (ICU) is relatively sparse and results vary [6, 14, 21, 22]. Among other factors, variation can be attributed to the wide variety of LUS methodologies used [23]. Proposed techniques include scoring aeration patterns [14, 24, 25] and counting the number of B-lines [22, 26]. The range of examined thoracic regions varies from 4 to 28 zones [13, 14, 21, 23, 26, 27]. Simplified scores offer appeal for clinical use, which is offset by a potential loss of information. Comprehensive scoring methods may provide higher accuracy at the cost of an extended examination time [28]. There is a need for studies that compare different proposed LUS scores to assess pulmonary edema in ARDS, keeping in mind the tension between accuracy and clinical applicability.

In this study, the primary outcome was the correlation of four existing LUS scores with EVLWi as the reference standard. Secondary aims were to evaluate the correlation of the change in LUS scores and EVLWi between two time points, and to assess the diagnostic accuracy of LUS scores for severe pulmonary edema defined as an EVLWi > 15 ml/kg [17]. We hypothesized that both extended and limited LUS aeration scores can quantify PiCCO-derived pulmonary edema and changes therein in patients with COVID-19 ARDS.

## Methods

### Study design and ethical considerations

This study was a predefined secondary analysis of data collected within the multicenter, randomized, double-blind, placebo-controlled InventCOVID trial (The efficacy and safety of intravenous imatinib in invasively ventilated patients with COVID-19-related acute respiratory distress syndrome, ClinicalTrials.gov identifier: NCT04794088) conducted between March 2021 and March 2022. The trial included invasively ventilated patients on mixed medical and surgical intensive care units (ICUs) at four hospitals in the Netherlands. Of these, two participating centers performed LUS. The Institutional Review Board of the Amsterdam UMC, location VUMC (identifier 2020.0752) approved the study and written informed consent for the use of clinical data, LUS imaging and blood samples was obtained from the patient or their legal representatives.

### Eligibility

All data were obtained from patients enrolled in the InventCOVID trial. Patients were included in the current study if aged ≥ 18 years, classified as moderate or severe ARDS [29] due to COVID-19, and in whom LUS and EVLWi measurements were performed at time point 1 (the day of enrollment into the InventCOVID trial). The main exclusion criteria for this study were missing LUS and EVLWi measurements at timepoint 1 or ≥ 4 missing regions on LUS exam. For a complete list of in- and exclusion criteria of the InventCOVID trial, we refer to the original work [30] and to Additional file 1 (p. 1).

### Measurements

EVLWi measurement by transpulmonary thermodilution was used as the reference test. The PiCCO catheter was placed into the femoral or brachial artery and the injectate temperature sensor was attached to the most proximal port of the central venous catheter. The cardiac output measurement was calibrated using transpulmonary thermodilution (PiCCO System, version 4.1; Pulsion Medical Systems; Munich, Germany). 20 ml of cold (< 8 °C) 0.9% saline solution was injected to cause a change in temperature of ≥ 0.2 °C at the arterial catheter tip. This procedure was repeated three times and the result was averaged to obtain the cardiac output. The volume of EVLW obtained from the PiCCO measurement performed by trained ICU nursing staff blinded for the index test. EVLW was indexed to predicted body weight to obtain EVLWi.

**Fig. 1:**
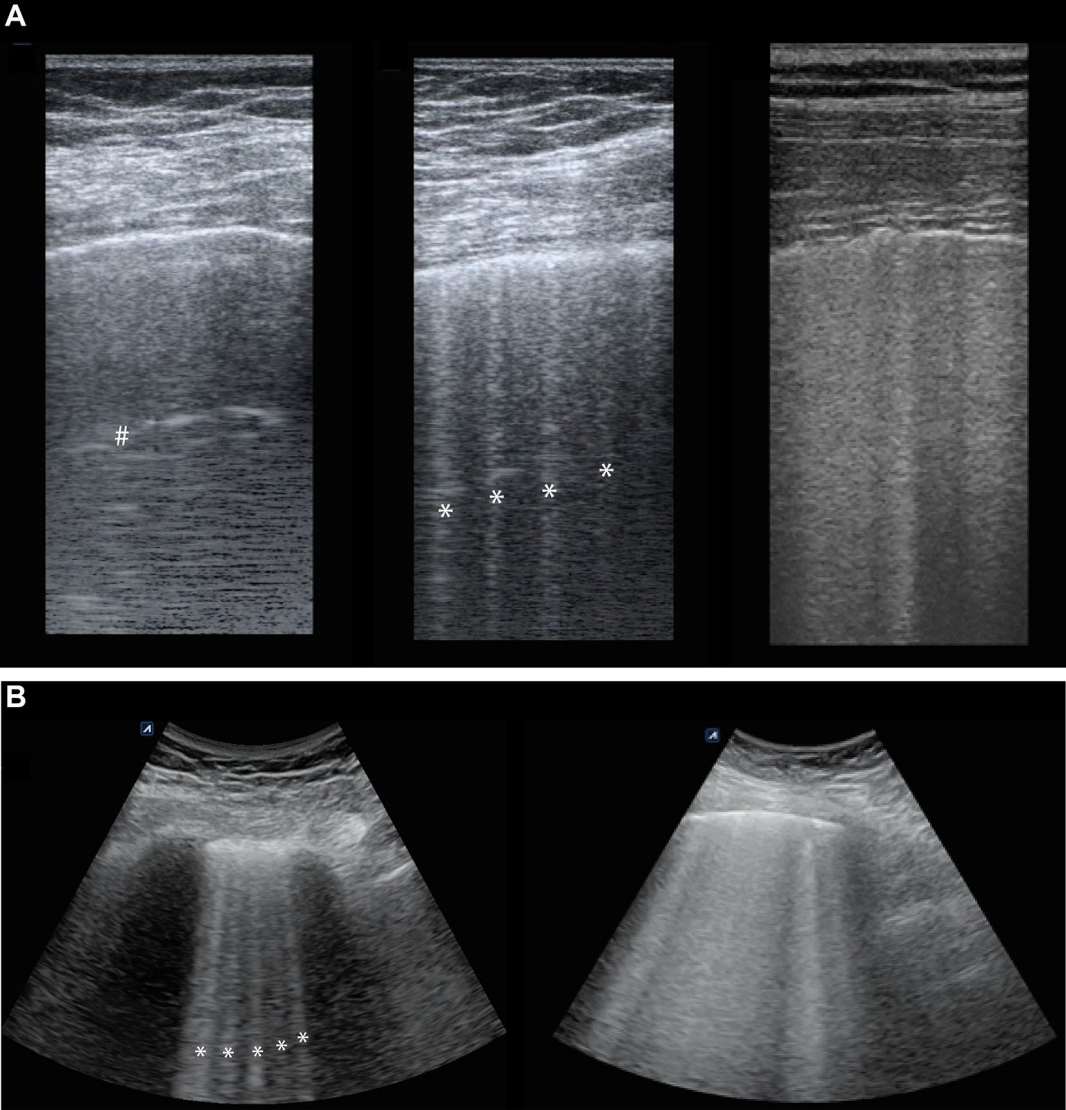
Lung ultrasound images used for aeration and B-line count scores. **A**: Example of lung ultrasound (LUS) images used for 8- and 12-region aeration scores using linear array probe (5.0–12.0 MHz). Left image: a line indicated by # (A pattern, 0 points); center image: ≥ 3 well-spaced B-lines indicated by * (B1 pattern, 1 point) with thickened pleura; right image: coalescent B-lines (B2 pattern, 2 points) with irregular, fragmented pleura. **B**: example of LUS images used for the B-line score using curved array probe (2.5–5.0 MHz). Left image: five B-lines, resulting in 5 points; right image: confluent B-lines taking up 100% of the intercostal space, resulting in 8 points. LUS  lung ultrasound

The index test for this study was LUS. LUS was performed using the LOGIQ-e (GE Healthcare, Milwaukee, USA), E-Cube i7/8 (Alpinion Medical Systems, Seoul, Republic of Korea) and Sonosite Edge II (Fujifilm Sonosite Inc., Bothell, USA) ultrasound machines. Prior to the start of this study, two LUS investigators (LNA, JS) were trained by two experienced ultrasonographers (MRS, MEH). All LUS images were obtained and scored offline by one of the two LUS investigators (LNA, JS) before retrieving the EVLWi measurement. The procedure of acquiring LUS images and determining the global LUS score has been previously described [9, 31]. In short, scanning in oblique orientation (i.e., length of the probe parallel to the costae), a linear array transducer (5.0–12.0 MHz) was used to examine two ventral, two lateral and two dorsal images per hemithorax, resulting in a 12-region scan. For the B-line score, images obtained with the curved array transducer were used (2.5–5.0 MHz) to reproduce the previously described method used for this score [18]. Harmonics were turned off to allow for optimal visualization of ultrasonographic artifacts and image depth was set at > 6 cm. Focus was adjusted to the height of the pleura. Figure 1 shows examples of LUS images used for scoring.

To obtain the global LUS score, LUS–ARDS and anterior–lateral score, loss of aeration was scored per region as previously described [9] and summarized in Table 1. To determine the LUS–ARDS score, a formula was developed by Smit et al. [25] based on a logistic regression model (see Table 1). The ∆LUS scores and ∆EVLWi were calculated by subtracting the measurement performed at time point 1 from the measurement at time point 2.

**Table 1:** Lung ultrasound scoring methods

LUS score	Regions scanned per hemithorax (total)	Points attributed for	Score calculation	Score range
Global LUS	6 (12)	LUS aeration patterns*	Sum of points assigned per region	0–36
LUS–ARDS	6 (12)	LUS aeration patterns* and pleural abnormalities**	2.5 × left hemithorax aeration score + 1 × right hemithorax aeration score + 3.5 × number of antero-lateral pleural abnormalities	0–91
Anterior–lateral	4 (8)	LUS aeration patterns*	Sum of points assigned per region	0–24
B-line	2 (4)	Number of counted B-lines	Sum of points assigned for the number of B-lines***	0–32

^*^0 points = A lines or < 3 B-lines (A pattern), 1 point =  ≥ 3 well-spaced B-lines occupying < 50% of the intercostal space (B1 pattern), 2 points = coalescent B-lines occupying > 50% of the intercostal space (B2 pattern) and 3 points = lung consolidation > 2 cm in diameter (C pattern)

^**^ Pleural abnormalities = thickened, fragmented and/or irregular pleura

^***^ See also Additional file 1: Table S1

*LUS* Lung ultrasound, *ARDS*  acute respiratory distress syndrome

### Statistical analysis

Categorical data were expressed as numbers and percentages and differences were tested using the Chi-square test. Continuous data were expressed as mean ± standard deviation (SD) or median ± interquartile range [IQR] and differences were analyzed depending on parametric or non-parametric distribution using a *t* test or one-way ANOVA, or a Mann–Whitney *U* or Kruskal–Wallis test, respectively. Tests were two-sided with a significance level of 0.05. Based on previous studies, a sample size of 26 or more was required for a correlation coefficient of 0.5 at an alpha of 0.05 and a power of 80% [6, 21].

To examine the association of the LUS scores with EVLWi, we performed Pearson correlation analysis. We tested for moderation of the association by positive end-expiratory pressure (PEEP) in a linear regression model. Diagnostic accuracy of the LUS scores for severe pulmonary edema was quantified using the area under the receiver operating characteristic curve (AUROCC) with a 95% confidence interval (CI). AUROCCs were compared using the De Long test. LUS score cutoffs were chosen based on a sensitivity of 90% or higher. This cutoff was chosen based on presumed clinical significance of a test with high sensitivity for identifying patients who may be at risk of developing severe pulmonary edema and may thus benefit from early intervention and monitoring. Sensitivity, specificity, positive predictive value (PPV) and negative predictive value (NPV) were calculated for these cutoffs. All statistical analyses were conducted using R studio, version 4.0.3.

## Results

### Patient characteristics

**Fig. 2:**
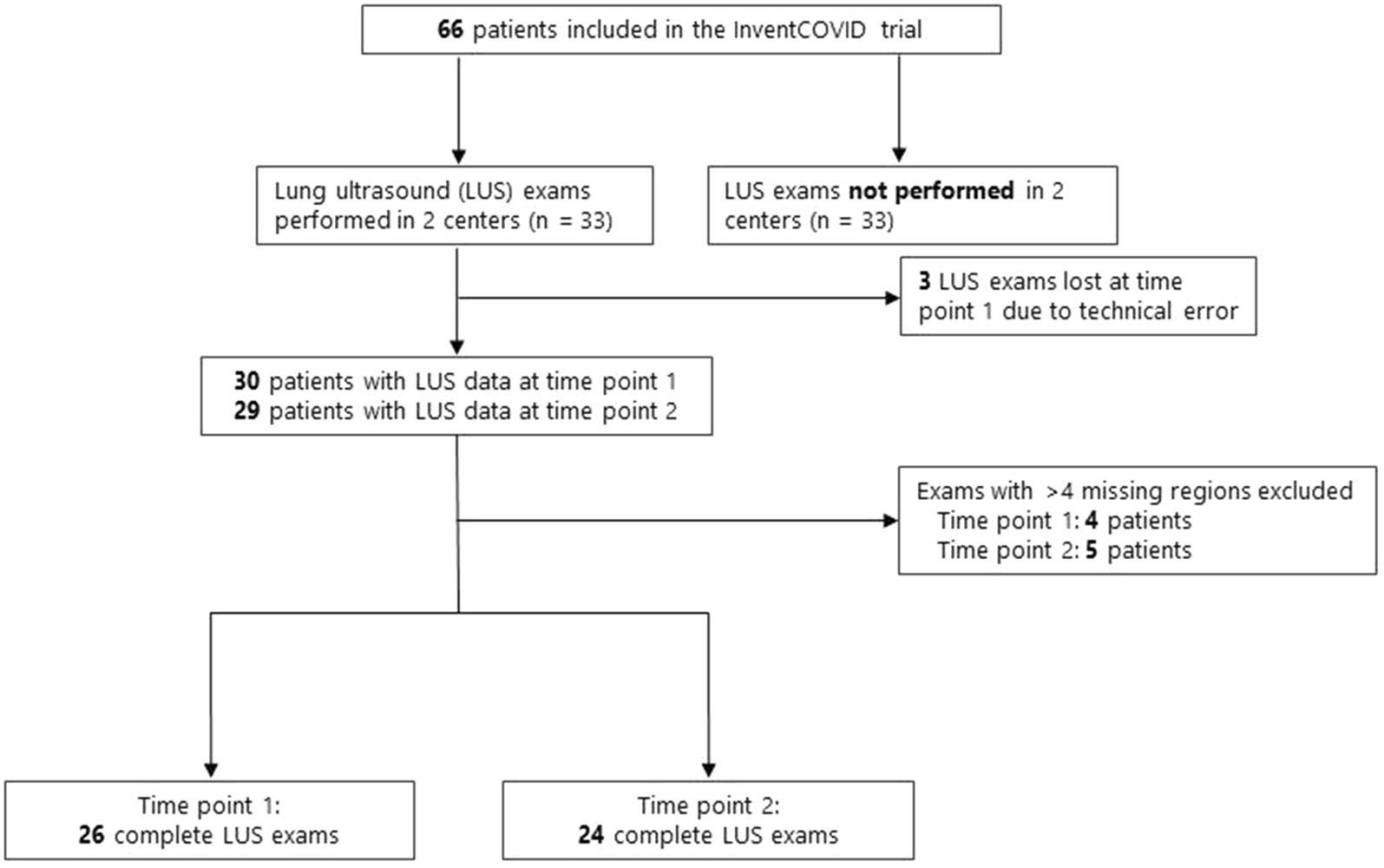
Flowchart of the inclusion and exclusion process. Due to logistic reasons, lung ultrasound (LUS) was only performed in 2 of the 4 centers participating in the InventCOVID trial (Amsterdam UMC, locations AMC and VUMC), resulting in LUS exams performed in 33 patients. Of these, 3 LUS exams were lost due to image recording errors. After the exclusion of exams missing > 4 regions, 26 patients had LUS data on study day 1 (= time point 1, within 48 hours after intubation) and 24 patients had LUS data on study day 4 (= time point 2). InventCOVID trial: the efficacy and safety of intravenous imatinib in invasively ventilated patients with moderate-to-severe COVID-19-related ARDS

**Fig. 3:**
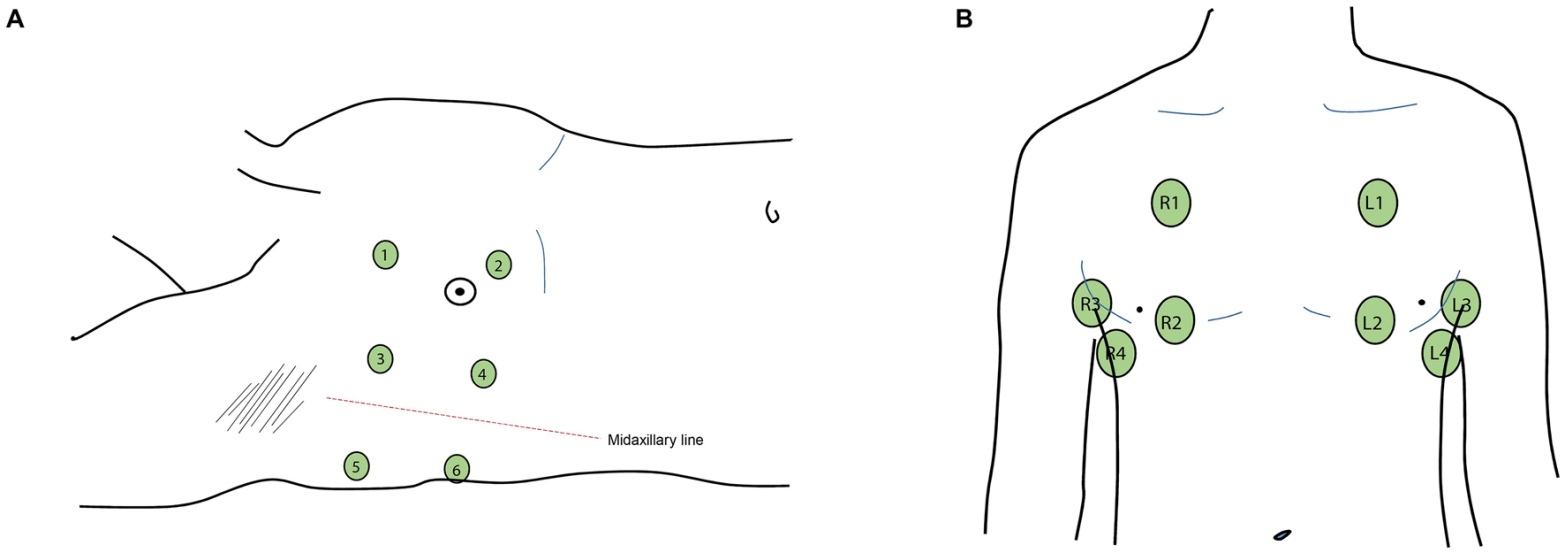
Regions of ultrasound examination. **A**: Figure depicting the positions for lung ultrasound (LUS) examination of the anterior (points 1 and 2), lateral (points 3 and 4) and posterior (points 5 and 6) regions (example using the right hemithorax). Regions were used for obtaining images for the global LUS score (6 regions per hemithorax, score range 0–36 points) and LUS–ARDS score (6 regions per hemithorax, score range 0–91 points). **B**: Ventral view of the thorax, depicting the 8 regions used for the anterior–lateral score (4 regions per hemithorax, score range 0–24 points). The four anterior points (R1, R2, L1, L2) were used for the aggregation of the B-line count score (2 regions per hemithorax, score range 0–32 points)

Demographic and clinical patient characteristics at timepoint 1 are summarized in Table 2 and the inclusion flow chart is depicted in Fig. 2. At timepoint 1, LUS examination was available in 30 (91%) out of 33 patients in whom LUS was performed and EVLWi data were available in 31 patients (94%). At timepoint 2, 29 (89%) out of 33 patients had available LUS and EVLWi data. After the exclusion of exams with > 4 missing regions, 26 (87%) of the 30 patients remained at timepoint 1 and 24 (83%) of 29 patients at timepoint 2 (Fig. 2, Additional file 1: Table S2). The majority of patients was classified as having moderate ARDS according to the Berlin criteria (74%, Table 2). A median EVLWi of 14.5 ml/kg with a pulmonary vascular permeability index (PVPi) of > 3 indicated moderate-to-severe permeability-driven pulmonary edema in this population (Table 2, Fig. 3).

**Table 2:** Demographic and clinical patient characteristics at time point 1

n	27
Admission characteristics	
Age in years, mean (SD)	65 (9.8)
Male sex, n (%)	12 (44.4)
BMI in kg/m^2^, median [IQR]	29 [26, 33]
ARDS classification (Berlin criteria)	
Severe, n (%)	7 (26)
Moderate, n (%)	20 (74)
Days since onset of COVID-19 symptoms, mean (SD)	11.88 (5.31)
SOFA score, median [IQR]	7 [7, 8]
Charlson comorbidity score, median [IQR]	2 [2, 3.5]
Cumulative fluid balance (24 h) in liters, median [IQR]	− 0.11 [− 0.59, 0.23)
IL-6 receptor inhibitors^1^, n (%)	23 (85)
Dexamethasone, n (%)	25 (93)
Comorbidities^2^	
COPD, n (%)	0 (0)
Heart failure, n (%)	0 (0)
Renal failure, n (%)	0 (0)
Myocardial infarction, n (%)	1 (3.7)
Ventilation and gas exchange	
PaO_2_ in kPa, mean (SD)	9.01 (1.36)
PaCO_2_ in kPa, mean (SD)	5.53 (1.04)
TV/PBW in ml/kg, median [IQR]	6.1 [5.7, 8.6]
PaO_2_/FiO_2_ in mmHg, mean (SD)	136 (40)
PEEP in cmH_2_O, median [IQR]	10 [8, 12]
Laboratory measurements	
Hemoglobin in mmol/L, median [IQR]	8.3 [7.7, 8.8]
Leucocytes × 10^9^/L, median [IQR]	10.8 [8.6, 15.0]
Thrombocytes × 10^9^/L, median [IQR]	320 [259, 385]
D-dimer in mg/L, median [IQR]	3.0 [1.6, 5.4]
Creatinine in micromol/L, median [IQR]	91 [62, 111]
NTproBNP in pg/ml, median [IQR]	180 [116, 424]
PiCCO measurements	
EVLW in ml, median [IQR]	1026 [843, 1176]
EVLWi in ml/kg, median [IQR]	14.5 [12.8, 17.1]
PVPI, median [IQR]	3.2 [2.8, 4.1]

^1^Tocilizumab (8 mg/kg single intravenous administration) or sarilumab (400 mg single intravenous administration) administered upon Intensive Care Unit admission; ^2^Known history of the disease at the moment of randomization

*ARDS* Acute Respiratory Distress Syndrome, *ALT*  alanine transaminase, *AST*  aspartate transaminase, *BMI*  Body Mass Index, *COPD*  chronic obstructive pulmonary disease, *COVID-19*  Coronavirus disease 2019, *EVLW(i)*  extravascular lung water (index), *FiO*_*2*_  fraction of inspired oxygen, *ICU* intensive care unit, *IL-6* = interleukin-6, *IQR*  interquartile range, *NTproBNP* N-terminal pro hormone brain natriuretic peptide, *PaO*_*2*_  partial pressure of oxygen, *PCR*  Polymerase chain reaction, *PEEP*  positive end-expiratory pressure, *PiCCO*  pulse contour cardiac output, *PVPI*  pulmonary vascular permeability index. *QT*_*c*_  corrected QT interval time, *SARS-CoV-2*  Severe acute respiratory syndrome coronavirus 2, *SD*  standard deviation, *SOFA*  Sequential Organ Failure Assessment, *TV/PBW*  tidal volume indexed to predicted body weight, *PVPI*  pulmonary vascular permeability index

### Correlation of LUS scores with EVLWi

**Fig. 4:**
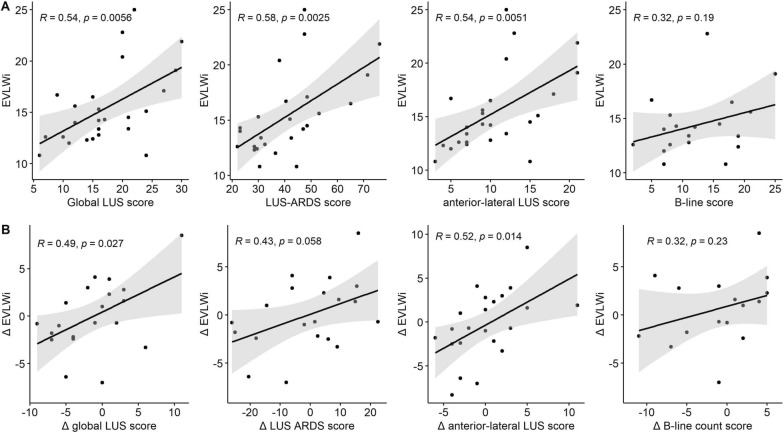
Correlation of LUS scores with EVLWi. **A**: Scatterplots depicting the correlation of the global lung ultrasound (LUS) score, LUS–ARDS score, the antero-lateral LUS score and B-line count score with the extravascular lung water index (EVLWi) at time point 1. **B**: Scatterplots depicting the correlation of the change (∆) in global LUS score, LUS–ARDS score, the antero-lateral LUS score and B-line count score with ∆EVLWi between time points 1 and 2. Number of data points in panel B differ from panel A, as missing data at timepoint 1 and/or 2 prevented calculation of ∆LUS score/∆EVLWi in several cases

The correlations of the LUS scores with EVLWi and ∆LUS scores with ∆EVLWi are depicted in Fig. 4A. The global LUS score and LUS–ARDS score both significantly correlated with EVLWi (Fig. 4A). The ∆global LUS score was significantly associated with ∆EVLWi between timepoints 1 and 2, while the correlation of the ∆LUS–ARDS score with ∆EVLWi did not reach statistical significance (Fig. 4B). Testing for moderation, there was no significant interaction between PEEP and the association between the global LUS score (*p* = 0.66), the LUS–ARDS score (*p* = 0.88) and the anterior–lateral LUS score (*p* = 0.46) with EVLWi.

Next, we examined the correlation of the LUS aeration score limited to the 8 anterior–lateral regions. The association with EVLWi (Fig. 4A) and the correlation of the ∆anterior–lateral LUS score and ∆EVLWi (Fig. 4B) were significant and comparable to the associations of the 12-region global LUS score with EVLWi. The B-line score and ∆B-line score did not significantly correlate with EVLWi (Fig. 4A) and ∆EVLWi (Fig. 4B), respectively.

### Diagnostic accuracy for EVLWi > 15 ml/kg

**Fig. 5:**
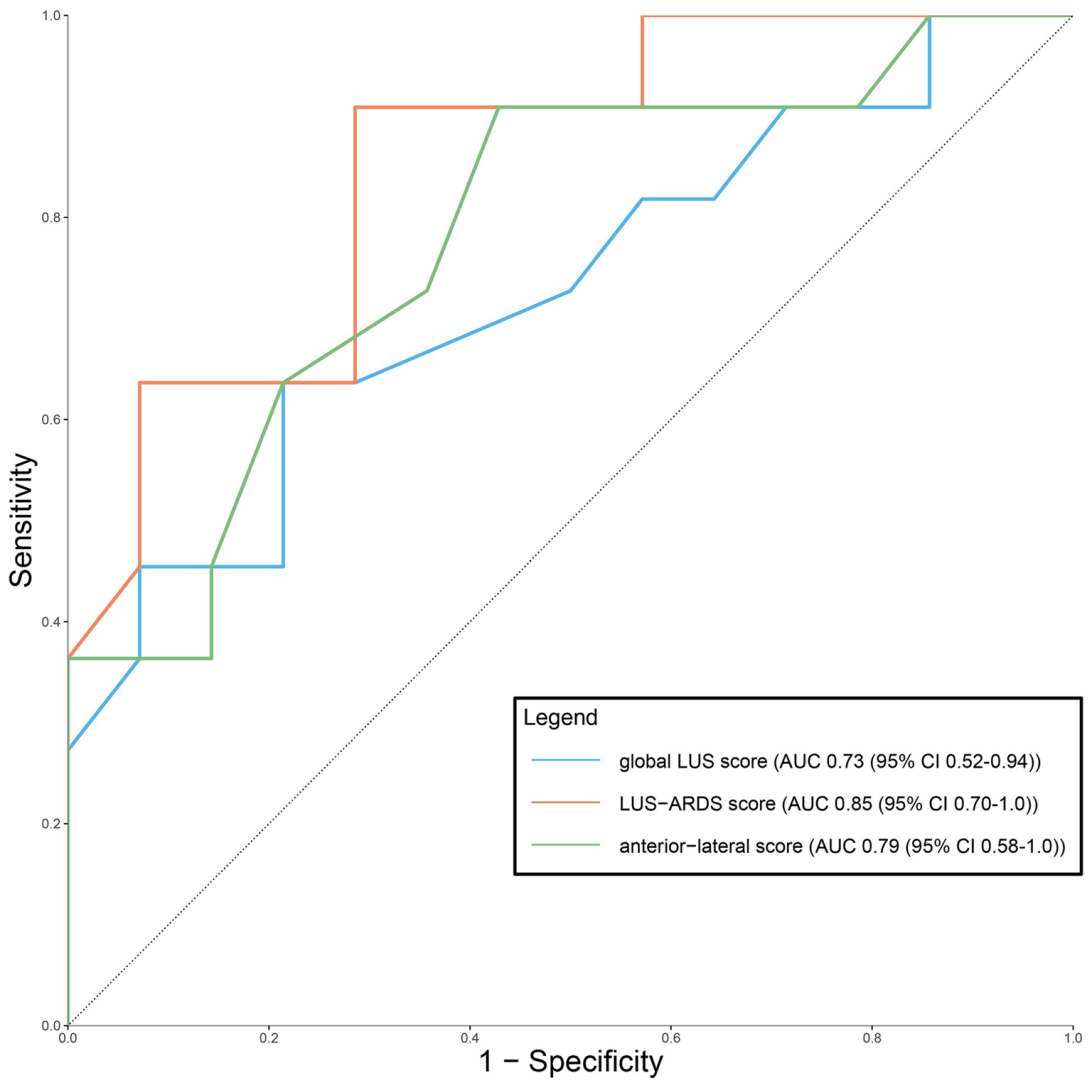
Diagnostic accuracy of the global lung ultrasound (LUS) score and the LUS–ARDS score for severe pulmonary edema. Receiver operating characteristics (ROC) curves for the prediction of severe pulmonary edema (extravascular lung water index > 15 ml/kg) by the global LUS score and the LUS–ARDS score. Comparison of the areas under the ROC curves (AUROCC) using the De Long test showed no statistically significant difference between the AUROCCs of the global LUS and LUS–ARDS score (*p* = 0.34), the global LUS and the anterior–lateral LUS score (*p* = 0.70) and the LUS–ARDS and the anterior–LUS score (*p* = 0.56)

Receiver operating characteristics (ROC) curves for the diagnostic accuracy of the LUS scores for detecting severe pulmonary edema (EVLWi > 15 ml/kg) are presented in Fig. 5. AUROCC, sensitivity, specificity, NPV and PPV are presented in Table 3 and the results of the De Long test comparing AUROCCs are displayed in the legend of Fig. 5.

**Table 3:** Diagnostic accuracy of lung ultrasound scores to detect EVLWi > 15 ml/kg

Score	Score cut off	AUROCC (95% CI)	Sensitivity (95% CI)	Specificity (95% CI)	PPV (95% CI)	NPV (95% CI)
Global LUS	11	0.73 (0.52–0.94)	0.91 (0.90–0.92)	0.29 (0.14–0.44)	0.50 (0.44–0.56)	0.80 (0.78–0.83)
LUS–ARDS	37	0.85 (0.70–1.00)	0.91 (0.90–0.92)	0.71 (0.67–0.76)	0.64 (0.59–0.69)	0.90 (0.89–0.91)
Anterior–lateral LUS	8	0. 79 (0.58–1.0)	0.91 (0.90–0.92)	0.57 (0.50–0.64)	0.63 (0.57–0.68)	0.89 (0.87–0.90)

Summary of the diagnostic accuracy measures of the 12-region global LUS and LUS–ARDS scores and the reduced 8-region anterior–lateral LUS score to detect severe pulmonary edema (EVLWi > 15 ml/kg) at the respective cutoff values. *ARDS*  Acute Respiratory Distress Syndrome, *AUROCC*  area under the receiver operating curve, *EVLWi*  extravascular lung water index, *LUS*  lung ultrasound, *NPV*  negative predictive value, *PPV*  positive predictive value

The global LUS score had an AUROCC of 0.73 (CI 0.52–0.94). A cutoff of 11 out of 36 points had a sensitivity of 0.91 and a specificity of 0.29 for severe pulmonary edema. The AUROCC of the LUS–ARDS score was 0.85 (CI 0.70–1.0), with a cutoff of 37 out of 91 points that resulted in a sensitivity of 0.91 and a specificity of 0.71. The anterior–lateral score had an AUROCC of 0.79 (CI 0.58–1.0). At cutoff of 8 of 24 points, the sensitivity was 0.91 and the specificity 0.57 (Table 3). Comparing the three AUROCCs using the De Long test showed no statistically significant differences between the global LUS and the LUS–ARDS score, the global LUS and the anterior–lateral score and the LUS–ARDS and the anterior–lateral scores (see legend Fig. 5).

## Discussion

In this predefined secondary analysis of the InventCOVID trial, we evaluated the correlation between four existing LUS scores and EVLWi in COVID-19 ARDS patients. The key findings of the study were: (1) the global LUS score, the LUS–ARDS score and the anterior–lateral score correlated with EVLWi, while the B-line score did not; (2) changes in the global LUS score and anterior–lateral score correlated with changes in EVLWi over time.

The 12- and 8-region scores examined in this study can quantify PiCCO-derived pulmonary edema measurements in COVID-19 ARDS. Combined with previous studies [24, 25, 32], our results further support the use of LUS for the assessment of pulmonary edema in patients with ARDS. The correlation of the shorter anterior-lateral score with EVLWi is in line with previous work that showed comparable performance of the 8-region score to more extensive protocols in assessing diagnostic accuracy and monitoring ARDS [33, 34]. The rationale of exempting the dorsal regions from examination is the prevalence of compression atelectasis and gravitational accumulation of pulmonary edema in the supine position [35]. Moreover, a score that requires less time to perform remains clinically attractive, as LUS is a bedside tool. Our data suggests that quantification of EVLW with the 8-region anterior–lateral score may be an alternative to the 12-region protocols to quantify pulmonary edema.

The performance of the LUS–ARDS score supports the score’s value in as an adjunct in the comprehensive assessment of patients with ARDS. Notably, this score was developed and validated for ARDS diagnosis [25] and not to predict pulmonary edema. Unlike other LUS aeration scores, the presence of pleural abnormalities contributes to the LUS–ARDS score. This choice was made to better capture the uncertain, non-binary nature of ARDS as a syndrome [36]. We hypothesize that taking into account pleural morphology in combination with the aeration score increases the likelihood of identifying severe pulmonary edema by functioning as an indicator of disease severity in the rest of the lung. Combined with the recently reported high accuracy for ARDS diagnosis [25], the score could be a useful adjunct to identify patients at risk of clinically relevant pulmonary edema. Validation in a non-COVID-19 ARDS cohort is needed to extrapolate our findings to the broader ARDS population.

To analyze the diagnostic accuracy of the LUS scores for detecting an EVLWi > 15 ml/kg, score cutoffs were chosen based on a sensitivity of > 90%. This comes at the expense of specificity—a choice which was made with clinical practice in mind. A clinician performing a LUS exam in a patient with ARDS can use a score below the determined cutoffs to rule out severe pulmonary edema at the moment of measurement. On the one hand, this may provide reassurance of the already implemented treatment. On the other hand, it can alert the clinician to monitor and/or to initiate proactive intervention in a patient who is clinically suspected to be at risk of deteriorating.

Considering the potential risk of over- or underestimation of pulmonary edema through the use of aeration patterns [33, 37–39], it follows that a score based solely on the number of B-lines may be more appropriate for focused quantification. Enghard et al. found an excellent correlation (*r* = 0.91) of a simplified 4-region B-line score with EVLWi in a mixed ICU population [22]. However, of the 50 patients, only 6 were classified as ARDS, considerably limiting the validity of their findings for the ARDS population. One study examined the same score in 26 ARDS patients and described a correlation (*r* = 0.66); however, it found that changes in B-line score could not predict variations in EVLWi [6]*.* In the current study, we found no significant correlation of the B-line score with EVLWi, nor with ∆EVLWi. Considering these discrepancies, it is questionable whether B-line counting is suitable for scoring pulmonary edema in ARDS patients. Reasons for the inconsistent performance of the score include that the choice of transducer and the interpretation of the sonographer significantly affect the reproducibility of this method [40].

Aside from assessing severity, monitoring changes in pulmonary edema and lung aeration is useful to evaluate treatment response. A change in global LUS score and the anterior–lateral LUS score between timepoints 1 and 2 was significantly associated with ∆EVLWi, and ∆LUS–ARDS score was positively associated with ∆EVLWi, despite not reaching statistical significance. Possibly, the global and anterior–lateral LUS scores are better suited to monitor pulmonary edema over time. A reason may be that the LUS–ARDS score considers pleural abnormalities, which may not be as sensitive to changes in EVLW as aeration patterns are. Based on the current findings and other studies [8, 41], LUS aeration scores seem useful to evaluate a change in EVLW in (COVID-19) ARDS. To validate this conclusion, a future study may include measurements at multiple timepoints.

The study has several strengths. First, the prospectively included population was exclusively comprised of patients with COVID-19 ARDS, making this a population with a single pulmonary etiology and thus providing a rare degree of relative homogeneity. Second, the availability of two timepoints of measurement allowed us to investigate the correlation of ∆LUS and ∆EVLWi, allowing for assessment of the value of LUS for monitoring pulmonary edema. Third, to our knowledge this is the first study to compare four previously proposed LUS scores that differ in terms of examined regions and/or means of score aggregation.

Some limitations should be acknowledged. The inclusion of COVID-19 ARDS patients with moderate-to-severe illness reduces external validity to ARDS populations with a different etiology or milder disease severity. However, we considered COVID-19 ARDS to be particularly suitable for this study, as it typically presents without the concomitant pathologies that challenge PiCCO measurement in other critically ill patients [42, 43]. Second, the study only included invasively ventilated patients, not patients receiving non-invasive modes, such as high-flow nasal oxygen. Therefore, we cannot draw conclusions about the use of LUS in a group that may particularly benefit from quantification of pulmonary edema [44, 45] and initiation of early intervention. Finally, the number of missing regions decreased the sample size and excluding patients with ≥ 4 missing regions may have induced a degree of selection bias.

This explorative study highlights that LUS can determine PiCCO-derived EVLWi, strengthening the rationale for its use to quantify pulmonary edema in patients with ARDS. Research into the application of LUS to quantify edema and use this information to guide adherence to a restrictive fluid balance is currently ongoing (ClinicalTrials.gov: NCT05188092). Yet, the results of this small study also underline the need for a larger sample, in which the different LUS techniques are systematically compared to the reference standard with the aim of drawing a definitive conclusion on the optimal score to be used in clinical practice.

In conclusion, both 12-region LUS scores and the 8-region anterior–lateral score correlated with PiCCO-derived pulmonary edema in invasively ventilated patients with COVID-19 ARDS. The anterior–lateral score seems to be as useful to quantify and monitor change in pulmonary edema as the 12-region scores. Combined with its recently reported high accuracy to diagnose ARDS, the LUS–ARDS score may be best-suited for a comprehensive assessment of ARDS diagnosis and pulmonary edema severity.

### Supplementary Information

**Additional file 1: Appendix 1. **In- and exclusion criteria of the InventCOVID trial. **Table S1**. The 4-region B-line count score. **Table S2. **Lung ultrasound scores per patient.

## Data Availability

All anonymized patient data will be available after the publication of the article. The data can be requested from the corresponding author (l.atmowhardjo@amsterdamumc.nl) by other researchers when reuse conditions are met.
