# Supplementary material for: The diagnostic accuracy of lung ultrasound to determine PiCCO-derived extravascular lung water in invasively ventilated patients with COVID-19 ARDS
Source: Ultrasound J. 2023 Oct 2;15:40. doi: 10.1186/s13089-023-00340-7 (PMC10545605; doi:10.1186/s13089-023-00340-7)
Supplement: Supplementary file 1 — Additional file 1: Appendix 1. In- and exclusion criteria of the InventCOVID trial. Table S1. The 4-region B-line count score. Table S2. Lung ultrasound scores per patient. [file 13089_2023_340_MOESM1_ESM.docx]

**Online supplement**

**Manuscript Title:** The diagnostic accuracy of lung ultrasound to determine PiCCO-derived extravascular lung water in invasively ventilated patients with COVID-19 ARDS

**Table of contents**

[**Appendix 1:** In- and exclusion criteria InventCOVID trial 1](#_Toc143849860)

[**Table S1:** The 4-region B-line count score 2](#_Toc143849861)

[**Table S2:** Lung ultrasound scores per patient 3](#_Toc143849862)

# **Appendix 1:** In- and exclusion criteria InventCOVID trial

**Inclusion criteria:**

- Age ≥18 years
- Moderate to severe ARDS, as defined by the Berlin definition for ARDS, intubated for invasive mechanical ventilation
- A PCR positive for SARS-CoV2 infection within the current disease episode
- Written informed consent provided by the patient or the patient’s legally authorized representative.

**Exclusion criteria:**

- Persistent septic shock (> 24 h) with a mean arterial pressure ≤ 65 mmHg and serum lactate level > 4 mmol/L (36 mg/dL) despite adequate volume resuscitation and vasopressor use (norepinephrine > 0·2 μg/kg/min) for > 6 h;
- Pre-existing chronic pulmonary disease, including known diagnosis of interstitial lung disease; known diagnosis of chronic obstructive pulmonary disease GOLD Stage IV or forced expiratory volume in 1 s < 30% predicted; diffusing capacity for carbon monoxide < 45% (if test results are available); total lung capacity < 60% of predicted (if test results are available);
- Chronic home oxygen treatment;
- Pre-existing heart failure with known left ventricular ejection fraction < 40%;
- Active treatment of hematological or non-hematological cancer with targeted immuno- or chemotherapy, or thoracic radiotherapy in the last year;
- Currently receiving extracorporeal life support;
- Severe chronic liver disease with Child-Pugh score > 12;
- Subjects in whom a decision to withdraw medical care is made (e.g., palliative setting);
- Inability of the ICU staff to initiate investigational medicinal product administration within 48 h of intubation;
- Known to be pregnant or breast-feeding;
- Enrolled in a concomitant clinical trial of an investigational medicinal product;
- White blood cell count < 2·5 × 10^9^/l; hemoglobin < 4·0 mmol/l or thrombocytes < 50 × 10^9^/l;
- The use of strong CYP3A4 inducers, including the following drugs: Carbamazepine, efavirenz, enzalutamide, phenobarbital, phenytoin, hypericum (St. John’s wort), mitotane, nevirapine, primidone, rifabutin, rifampicin;
- The presence of an intra-aortic balloon pump (IABP);
- Known medical history of aortic aneurysm in the trajectory of the PiCCO measurement between central venous line and arterial detector;
- Known medical history of an intracardiac shunt.

# **Table S1:** The 4-region B-line count score

| **Ultrasound finding** | **Score** |
| --- | --- |
| No B-line/ICS^*^ | 0 |
| One B-line/ICS | 1 |
| Two B-lines/ICS | 2 |
| Three B-lines/ICS | 3 |
| Four B-lines/ICS | 4 |
| Five B-lines/ICS | 5 |
| Confluent B lines >50% ICS | 6 |
| Confluent B lines >75% ICS | 7 |
| Confluent B lines 100% ICS | 8 |

**ICS = intercostal space*

# **Table S2:** Lung ultrasound scores per patient

| **Patient** | **Global LUS 1** | **Global LUS 2** | ∆**global LUS** | **Anterior-lateral 1** | **Anterior-lateral 2** | ∆**anterior-lateral** | **B-line score 1** | **B-line score 2** | ∆**B-line score** | **LUS-ARDS 1** | **LUS-ARDS 2** | ∆**LUS-ARDS** | **Pleural abnorm. 1** | **Pleural abnorm. 2** |
| --- | --- | --- | --- | --- | --- | --- | --- | --- | --- | --- | --- | --- | --- | --- |
| 1 | 16 | 7 | -9 | 10 | 4 | -6 | 18 | 4 | -14 | 45 | 6.5 | -38.5 | 3 | 0 |
| 2 | 16 | 9 | -7 | 9 | 3 | -6 | 8 | 3 | -5 | 30 | 5 | -25 | 2 | 0 |
| 3 | 22 | *NA* | *NA* | 12 | 8 | -4 | *NA* | *NA* | *NA* | 47.5 | *NA* | *NA* | 5 | 3 |
| 4 | 20 | 13 | -7 | 12 | 8 | -4 | *NA* | *NA* | *NA* | 38 | 44 | 6 | 4 | 4 |
| 5 | 10 | 11 | 1 | 7 | 8 | 1 | 8 | 13 | 5 | 22 | 26.5 | 4.5 | 1 | 2 |
| 6 | 15 | 10 | -5 | 7 | 7 | 0 | 19 | 23 | 4 | 29.5 | 44.5 | 15 | 2 | 7 |
| 7 | 16 | *NA* | *NA* | 10 | *NA* | *NA* | 12 | NA | NA | 47 | *NA* | *NA* | 4 | 0 |
| 8 | 12 | 12 | 0 | 9 | 8 | -1 | 21 | 20 | -1 | 53 | 45 | -8 | 8 | 7 |
| 9 | 7 | *NA* | *NA* | 6 | *NA* | *NA* | 2 | *NA* | *NA* | 28.5 | *NA* | *NA* | 5 | 0 |
| 10 | 14 | 8 | -6 | 4 | 4 | 0 | *NA* | *NA* | *NA* | 28.5 | 26.5 | -2 | 3 | 5 |
| 11 | 11 | 2 | -9 | 5 | 1 | -4 | 7 | 7 | 0 | 36.5 | 10.5 | -26 | 3 | 2 |
| 12 | 15 | 14 | -1 | 10 | 9 | -1 | 18 | 9 | -9 | 65 | 59 | -6 | 11 | 8 |
| 13 | 6 | 9 | 3 | 3 | 3 | 0 | 7 | 1 | -6 | 30.5 | 24.5 | -6 | 5 | 3 |
| 14 | 16 | 12 | -4 | 7 | 8 | 1 | 19 | 8 | -11 | 42.5 | 45 | 2.5 | 7 | 6 |
| 15 | 12 | 13 | 1 | 7 | 10 | 3 | 7 | 12 | 5 | 23 | 29.5 | 6.5 | 2 | 1 |
| 16 | 9 | 8 | -1 | 5 | 3 | -2 | 5 | 4 | -1 | 40.5 | 42 | 1.5 | 7 | 7 |
| 17 | 24 | *NA* | *NA* | 16 | *NA* | *NA* | *NA* | *NA* | *NA* | 42 | *NA* | *NA* | 0 | 0 |
| 18 | 21 | 32 | 11 | 12 | 17 | 5 | 11 | 15 | 4 | 31 | 47 | 16 | 0 | 0 |
| 19 | 17 | 15 | -2 | 9 | 11 | 2 | 9 | 8 | -1 | 23 | 38.5 | 15.5 | 0 | 1 |
| 20 | 21 | 17 | -4 | 15 | 12 | -3 | 16 | 18 | 2 | 48.5 | 30.5 | -18 | 1 | 0 |
| 21 | 24 | 24 | 0 | 15 | 12 | -3 | 17 | 19 | 2 | 44.5 | 30 | -14.5 | 0 | 0 |
| 22 | 16 | 19 | 3 | 10 | 15 | 5 | 11 | 12 | 1 | 32.5 | 42 | 9.5 | 1 | 2 |
| 23 | 30 | 25 | -5 | 21 | 18 | -3 | *NA* | 15 | *NA* | 76 | 55.5 | -20.5 | 5 | 3 |
| 24 | 20 | *NA* | *NA* | 13 | 24 | 11 | 14 | NA | *NA* | 47.5 | *NA* | *NA* | 1 | 1 |
| 25 | 29 | 35 | 6 | 21 | 23 | 2 | 25 | 18 | -7 | 71.5 | 80.5 | 9 | 4 | 7 |
| 26 | 27 | 29 | 2 | 18 | 21 | 3 | *NA* | *NA* | *NA* | 48.5 | 71 | 22.5 | 1 | 5 |

*LUS = lung ultrasound; ‘1’ behind a score indicates time point 1; ‘2’ behind a score indicates time point 2; pleural abnorm. = number of pleural abnormalities in the anterior-lateral regions*
